# Construction of LncRNA-Related ceRNA Networks in Longissimus Dorsi Muscle of Jinfen White Pigs at Different Developmental Stages

**DOI:** 10.3390/cimb46010022

**Published:** 2024-01-02

**Authors:** Shouyuan Wang, Mingyue Shi, Yunting Zhang, Jin Niu, Wenxia Li, Jiale Yuan, Chunbo Cai, Yang Yang, Pengfei Gao, Xiaohong Guo, Bugao Li, Chang Lu, Guoqing Cao

**Affiliations:** College of Animal Science, Shanxi Agricultural University, No. 1 Mingxian South Road, Taigu 030801, China; s20222346@stu.sxau.edu.cn (S.W.); sxndsmy@163.com (M.S.); hahahaha0306@163.com (Y.Z.); niu-jin302@163.com (J.N.); lwx8lois@163.com (W.L.); yunjiale0248@163.com (J.Y.); caichunbo@sxau.edu.cn (C.C.); yangyangyh@sxau.edu.cn (Y.Y.); gpf800411@sxau.edu.cn (P.G.); xhguo@sxau.edu.cn (X.G.); bugaoli@sxau.edu.cn (B.L.)

**Keywords:** Jinfen White pig, longissimus dorsi, ceRNA, regulation networks

## Abstract

The development of skeletal muscle in pigs might determine the quality of pork. In recent years, long non-coding RNAs (lncRNAs) have been found to play an important role in skeletal muscle growth and development. In this study, we investigated the whole transcriptome of the longissimus dorsi muscle (LDM) of Jinfen White pigs at three developmental stages (1, 90, and 180 days) and performed a comprehensive analysis of lncRNAs, mRNAs, and micro-RNAs (miRNAs), aiming to find the key regulators and interaction networks in Jinfen White pigs. A total of 2638 differentially expressed mRNAs (DE mRNAs) and 982 differentially expressed lncRNAs (DE lncRNAs) were identified. Compared with JFW_1d, there were 497 up-regulated and 698 down-regulated DE mRNAs and 212 up-regulated and 286 down-regulated DE lncRNAs in JFW_90d, respectively. In JFW_180d, there were 613 up-regulated and 895 down-regulated DE mRNAs and 184 up-regulated and 131 down-regulated DE lncRNAs compared with JFW_1d. There were 615 up-regulated and 477 down-regulated DE mRNAs and 254 up-regulated and 355 down-regulated DE lncRNAs in JFW_180d compared with JFW_90d. Compared with mRNA, lncRNA has fewer exons, fewer ORFs, and a shorter length. We performed GO and KEGG pathway functional enrichment analysis for DE mRNAs and the potential target genes of DE lncRNAs. As a result, several pathways are involved in muscle growth and development, such as the PI3K-Akt, MAPK, hedgehog, and hippo signaling pathways. These are among the pathways through which mRNA and lncRNAs function. As part of this study, bioinformatic screening was used to identify miRNAs and DE lncRNAs that could act as ceRNAs. Finally, we constructed an lncRNA–miRNA–mRNA regulation network containing 26 mRNAs, 7 miRNAs, and 17 lncRNAs; qRT-PCR was used to verify the key genes in these networks. Among these, XLOC_022984/miR-127/ENAH and XLOC_016847/miR-486/NRF1 may function as key ceRNA networks. In this study, we obtained transcriptomic profiles from the LDM of Jinfen White pigs at three developmental stages and screened out lncRNA-miRNA-mRNA regulatory networks that may provide crucial information for the further exploration of the molecular mechanisms during skeletal muscle development.

## 1. Introduction

Approximately 40% of pigs’ body weight is made up of skeletal muscle, which is the primary source of meat production in the animal [1]. Skeletal muscle also performs a variety of biological functions, including locomotion, posture maintenance, heat generation, venous blood flow, and breathing control [2]. The growth and development of skeletal muscle is a complex biological process, affected by a variety of signaling pathways and regulatory factors [3], for example, myogenic regulatory factor families such as *MyoD*/*MyoD1*, *MyoG*, *Myf5*, and *MRF4*/*Myf6*, all of which are known transcription factors in muscle [4]. In addition, non-coding RNA (ncRNA) has received increasing attention in skeletal muscle development in recent years.

With the development of modern sequencing technologies, researchers have discovered that non-coding transcripts account for most of the genome [5]. Among these, microRNAs (miRNAs) and long non-coding RNAs (lncRNAs) play key roles in most biological processes, including skeletal muscle development [6]. Myogenic regulators such as miR-1 [7], miR-206 [8,9], and miR-133 [10] have been shown to be essential for muscle development. The regulation of gene function may also be mediated by lncRNAs through chromatin remodeling, DNA methylation, histone modification, and other mechanisms [11]. It also modulates transcription factor activity and forms triple helices with DNA, in addition to regulating adjacent genes’ transcription [12]. Furthermore, lncRNAs may also act as competing endogenous RNAs (ceRNAs) or “molecular sponges” to regulate myocardial fibrosis [13] and muscle differentiation [14]. Through this mechanism, lnc-133b/miR-133b/IGF1R promotes satellite cell proliferation [15], and lncRNA-ak017368 acts as a ceRNA of miR-30c to promote myoblast proliferation [16]. In addition, as a molecular sponge of miR-487b, lncMAR1 promotes skeletal muscle differentiation and regeneration by derepressing Wnt5a, which is an important regulator of myogenesis [17]. The ceRNA network is a complex post-transcriptional regulatory mechanism in cells, in which coding and non-coding RNAs competitively bind miRNAs to regulate the expression of target mRNAs.

There have been many studies using RNA-Seq to find important genes in the development of pigs. By using the transcriptomic data of the longissimus dorsi muscle (LDM) in Northeast Min pigs and Changbaishan wild boars, Xu identified a set of DEGs related to muscle fiber development [18]. In 2019, the genes related to the postnatal growth rate were identified by analyzing transcriptome and proteome data derived from prenatal muscle tissues of Tibetan pigs, Wujin pigs, and Large White pigs. Among these, 20 genes were found to have important roles in myoblast differentiation and muscle fiber formation [19]. In addition, RNA-Seq has been combined with newer technologies to further explore key factors. For example, by combining ATAC-seq with RNA-seq data, Miao identified several transcription factors, such as *ASNS* and *CARNS*1, that may be related to skeletal muscle growth and development in the LDM of Luchuan and Duroc pigs [20]. RNA-Seq was also used to identify lncRNAs in porcine skeletal muscle. Thousands of differentially expressed lncRNAs were identified in wild-type and gene-knockout Bama pigs using RNA-Seq [21]. RNA-Seq was used to identify 322 differentially expressed lncRNAs in the LDM of Beijing Black pigs and Yorkshire pigs [22]. Based on the above research, RNA-Seq was used to identify the transcriptome profile of Jinfen White pigs.

In this study, the transcriptome of the LDM of Jinfen White pigs was sampled at three growth stages (nine samples): 1 day (JFW_1d), 90 days (JFW_90d), and 180 days (JFW_180d). We investigated the different expressed genes in different groups and constructed the networks, which will allow us to identify important ncRNAs and their regulatory networks in the LDM of Jinfen White pigs. This will advance the knowledge of the ncRNA’s role in skeletal muscle development in pigs.

## 2. Materials and Methods

### 2.1. Animals and LDM Tissue Collection

A total of nine healthy Jinfen White pigs were selected from the Datong Pig Breeding Farm; three pigs were slaughtered at each of the following developmental stages: 1 day, 90 days, and 180 days after birth. All pigs were stunned with electricity to ameliorate the suffering of the pigs before death, followed by exsanguination using transverse incision of the neck. The longissimus dorsi muscle tissue was collected and stored at −80 °C. All animals were slaughtered following the procedures stipulated by the Animal Welfare and Ethics Committee of Shanxi Agricultural University (Approval No. SXAU-EAW-2021Sus.AB.0926001).

### 2.2. RNA Extraction and cDNA Synthesis

Total RNA extraction from LDM samples followed RNAiso Plus reagent (Takara, Kofu, Japan) protocol; 0.5 cm^3^ of tissue required 1 mL RNAiso Plus. Extracted RNA samples were measured with an ND-1000 (Thermo Fisher Scientific, Waltham, MA, USA) instrument. We synthesized cDNA using PrimeScript RT Reagent Kit with gDNA Eraser Kit (Takara, Kofu, Japan). For miRNAs, we performed cDNA synthesis according to the protocol of miRNA 1st Strand cDNA Synthesis Kit (Vazyme, Nanjing, China). Qualified samples were used for subsequent RNA-Seq.

### 2.3. Construction of cDNA Library and Illumina Sequencing

Ribosomal RNA was digested during the construction of the library, then the fragments were randomly divided into 250–300 bp. The cDNA library was prepared using the TruSeq Double-Strand Total RNA Library Prep Kit with random primers to generate fragmented RNA for the first-strand cDNA. For the second cDNA strand, the dNTP reagent in the dTTP was replaced by Dutp, resulting in the incorporation of A/U/C/G bases into the second cDNA strand. Following end repair and addition of poly-A tails, cDNA fragments of 150–200 bases were isolated, and single-stranded cDNA was obtained using uracil aza-glycosylase (UNG). Then, nine RNA-Seq libraries were generated. The data are publicly available in the NCBI GEO database with the accession number PRJNA867525. The sequencing was conducted on the Illumina Genome Analyzer II System at Beijing Novo-gene Bioinformatics Technology Co., Ltd. (Beijing, China), utilizing paired-end high-throughput sequencing.

### 2.4. Analysis of RNA-Seq Data

Each sample’s raw RNA-Seq reads were first processed to remove adapter sequences and low-quality reads. Two mismatches were allowed from each library when mapping to the pig reference genome (Sscrofa11.1) [23]. The mapped reads from each individual animal were assembled. Each gene was analyzed according to its fragments per kilobase of exons per million mapped reads (FPKM) [24]. We used R package GOseq to perform GO enrichment analysis of DEGs with the adjusted *p* value (*P*-adj) of 0.05 [25]. KEGG pathways were statistically enriched with DEGs using the KOBAS application (version 3.0) [26].

### 2.5. Identification of LncRNAs

We evaluated protein-coding potential by separating mRNAs from lncRNAs using CPC (Coding Potential Calculator) [27], CNCI (Coding-Non-Coding Index) [28], and CPAT (Coding Potential Assessment Tool) [29]. A genomic analysis was conducted on predicted lncRNAs to understand the differences between them and mRNAs [30].

### 2.6. Construction of LncRNA–miRNA–mRNA Networks

MiRanda (v3.3a) was used first to predict miRNA–lncRNA negative interactions [31]. Correlations between miRNA and mRNA and between miRNA and lncRNA were evaluated using Pearson correlation coefficients and *p* values. After that, the lncRNA–miRNA–mRNA interaction networks were constructed and visualized using Cytoscape (v3.5.1) [32]. RNAhybrid (https://bibiserv.cebitec.uni-bielefeld.de/rnahybrid, accessed on 7 May 2023) was used to predict the binding site secondary structure between miRNA and other sequences.

### 2.7. Validation of Sequencing Results Using Quantitative Real-Time PCR (qRT-PCR)

As a quantitative measurement of the reliability of sequencing data, qRT-PCR was performed to determine the expression level of 12 genes.

For mRNAs and lncRNAs, 18S rRNA was selected as the internal control. Following the manufacturer’s instructions, each 20 µL qRT-PCR reaction mixture contained 2 × SYBR Premix Ex Taq II (10 µL), cDNA (1.2 µL), and nuclease-free H_2_O (7.6 µL), along with 6 pmol each of the forward and reverse PCR primers. The following parameters were used for qRT-PCR: pre-denaturation for 30 s at 95 °C, then 40 cycles of 95 °C for 15 s, 60 °C for 30 s. The melting curve program was 95 °C 15 s, 60 °C 60 s, 95 °C 15 s.

For miRNAs, U6 snRNA was selected as the internal control. The primers for miRNAs were designed using the stem-loop approach. Each 20 µL qRT-PCR reaction mixture contained EvaGreen miRNA MasterMix (10 µL), cDNA (1 µL), nuclease-free H_2_O (7.8 µL), and 6 pmol each of the forward and reverse PCR primers. The following parameters were used for qRT-PCR: pre-denaturation for 10 min at 95 °C, then 40 cycles of 95 °C for 10 s, 63 °C for 15 s, and 72 °C for 8 s.

Relative expression levels of genes and miRNAs were calculated using the 2^−ΔΔCt^ method [33]. The primer sequences of genes are shown in the additional file (Table S1).

### 2.8. Statistical Analysis

Statistical analyses were performed using SPSS (v22.0). One-way ANOVA was used to compare the expression of mRNA, miRNA, and lncRNA at different stages. Differences with a *p* value of <0.05 were considered statistically significant.

## 3. Results

### 3.1. Landscape of the RNA Transcriptomes

From the nine LDM samples, a total of 909 million raw reads were obtained. In the JFW_1d group, an average of 101.37 million raw data were obtained and 100.73 million clean data were obtained after removing the adapter sequences and low-quality reads. In the JFW_90d group, an average of 99.38 million raw data and 98.70 million clean data were obtained. In addition, an average of 102.14 million raw data and 101.45 million clean data were obtained in JFW_180d. Moreover, the value of Q20 was above 97.81% for all samples, while the Q30 value was above 93.78%, indicating the high accuracy of the sequencing (Table 1).

### 3.2. Differentially Expressed mRNAs

**Figure 1:**
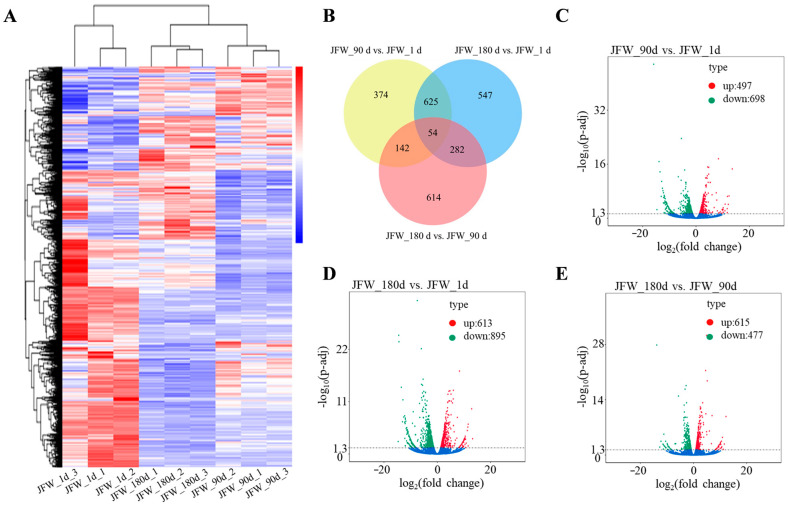
Expression of DE mRNAs. (**A**) Heatmap of DE mRNAs. (**B**) Venn diagram of DE mRNAs. (**C**–**E**) Volcano plots of DE mRNAs.

The reliability of the results was ensured by analyzing pairwise correlations based on normalized FPKM values; a large number of DE mRNAs were highly expressed in JFW_1d, whereas the expression patterns for DE mRNAs were similar between JFW_90d and JFW_180d and significantly different from those at JFW_1d (Figure 1A). A total of 2638 DE mRNAs were identified; 54 mRNAs were identified as differentially expressed in all comparisons, 1159 mRNAs were differently expressed in JFW_90d compared with JFW_1, 1508 mRNAs were differently expressed in JFW_180d compared with JFW_1d, and 1092 mRNAs were differently expressed in JFW_180d compared with JFW_90d (Figure 1B). Compared with JFW_1d, there were 497 up-regulated and 698 down-regulated DE mRNAs in JFW_90d, respectively (Figure 1C). In JFW_180d, there were 613 up-regulated and 895 down-regulated DE mRNAs compared with JFW_1d (Figure 1D). There were 615 up-regulated and 477 down-regulated DE mRNAs in JFW_180d compared with JFW_90d (Figure 1E).

### 3.3. Genomic Features of LncRNAs

**Figure 2:**
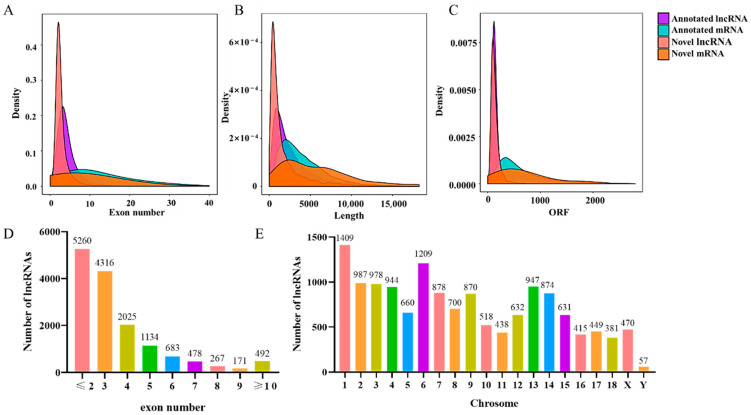
Genomic features of the identified lncRNAs and mRNAs. (**A**) Exon number distribution of mRNAs and lncRNAs. (**B**) Length distribution of mRNAs and lncRNAs. (**C**) ORF length distribution of mRNAs and lncRNAs. (**D**) Number of exons in lncRNAs. (**E**) Chromosomal distribution of lncRNAs.

Overall, 14,826 lncRNAs and 63,640 mRNAs were detected in all LDM samples. The analysis of protein-coding potential via CPC, CNCI, and CPAT revealed that 8432 of the lncRNAs were not considered capable of coding for proteins (Figure S1). As a result, we found that the lncRNAs contained a lower number of exons (mostly two or three exons) compared to the mRNAs (Figure 2A). The length of lncRNA transcripts was usually shorter than that of the mRNA (Figure 2B). The number of ORFs in lncRNA was also significantly less than that in mRNA (Figure 2C). Moreover, 5260 lncRNAs had only one or two exons, accounting for about 35% of the total identified lncRNAs (Figure 2D). However, the identified lncRNAs were distributed across all porcine chromosomes, mostly transcribed from chromosome 1 (1409) and with the lowest number (57) originating from the Y chromosome (Figure 2E).

### 3.4. Differentially Expressed lncRNAs

**Figure 3:**
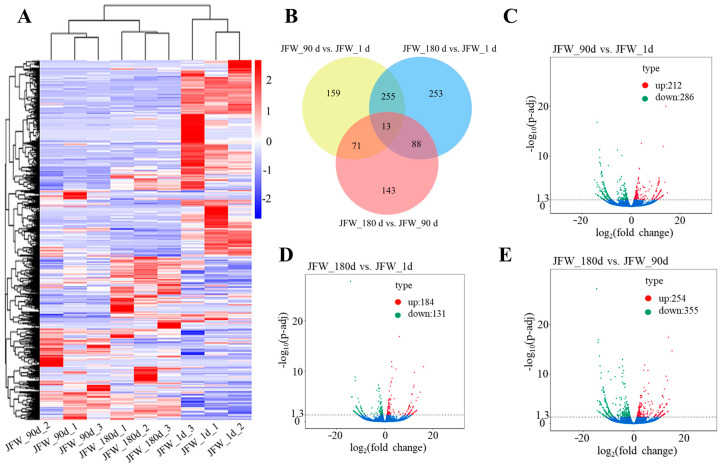
Expression of DE lncRNAs. (**A**) Heatmap of DE lncRNAs. (**B**) Venn diagram of DE lncRNAs. (**C**–**E**) Volcano plots of DE lncRNAs.

Similar to the expression pattern of DE mRNAs, the expression of DE lncRNAs in JFW_1d was significantly different from that in JFW_90d and JFW_180d (Figure 3A). In total, 982 DE lncRNAs were obtained in three periods of LDM, 498 DE lncRNAs were differently expressed in JFW_90d compared with JFW_1d, 315 DE lncRNAs were differently expressed in JFW_180d compared with JFW_1d, and 609 DE lncRNAs were differently expressed in JFW_180d compared with JFW_90d (Figure 3B). Compared with JFW_1d, there were 212 up-regulated and 286 down-regulated DE lncRNAs in JFW_90d (Figure 3C). There were 184 up-regulated and 131 down-regulated DE lncRNAs in JFW_180d compared with JFW_1d (Figure 3D). In JFW_180d, there were 254 up-regulated and 355 down-regulated DE lncRNAs compared with JFW_90d (Figure 3E).

### 3.5. Functional Enrichment Analysis

**Figure 4:**
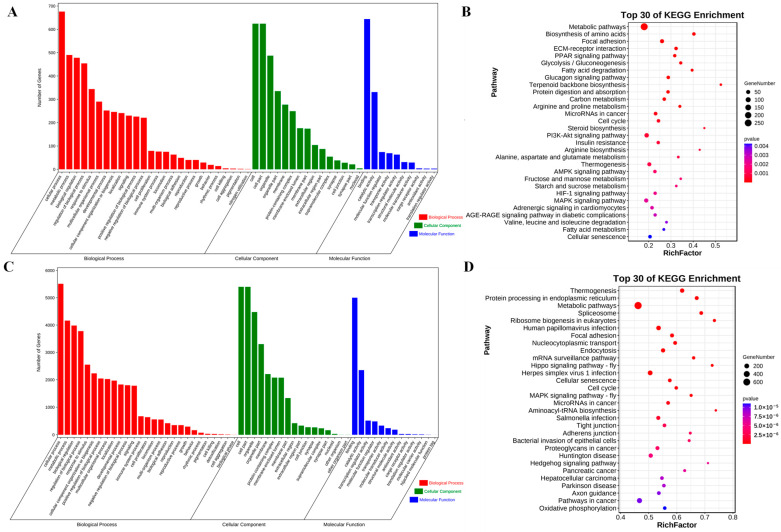
Functional enrichment analysis. (**A**) Results of GO enrichment for DE mRNAs. (**B**) Top 30 KEGG pathways with enrichment of DE mRNAs. (**C**) GO enrichment results for DE lncRNAs. (**D**) Top 30 KEGG pathways enriched for DE lncRNAs.

In order to investigate the potential functions of the observed DE mRNAs in the development of skeletal muscle, we performed GO and KEGG pathway enrichment analyses. GO annotation showed that the DE mRNAs mainly clustered in cellular processes and metabolism. They were enriched in 26 terms for biological processes, 15 terms for cell components, and 10 terms for molecular functions (Figure 4A). KEGG functional enrichment analysis revealed the top 30 significant pathways associated with the DE mRNAs (Figure 4B). These included several related to fat synthesis and metabolism, such as the PPAR, PI3K-Akt, and AMPK signaling pathways, as well as the pathways for insulin resistance and fatty acid metabolism. The enrichment results are shown in Supplementary Materials (Table S2).

The functions of DE lncRNAs were explored. Based on the correlation analysis of sequencing data, we investigated the target genes of lncRNAs. GO analysis revealed that there were 27, 17, and 13 GO terms in the biological process, cellular component, and molecular function categories (Figure 4C). Importantly, KEGG analysis revealed that the target genes of the DE lncRNAs were enriched on the hippo pathway, which is involved in myofibril assembly and myofiber growth [34]. Many DE lncRNA target genes were found to be enriched on the hippo pathway, such as lncRNA XLOC_016846, XLOC_016847, and XLOC_056276. Additionally, DE mRNAs and DE lncRNA target genes were both found to be enriched on the metabolic pathway and MAPK signaling pathways (Figure 4D). The target genes and enrichment results for DE lncRNAs are shown in Supplementary Materials (Table S3).

### 3.6. Construction of LncRNA–miRNA–mRNA Regulatory Networks

**Figure 5:**
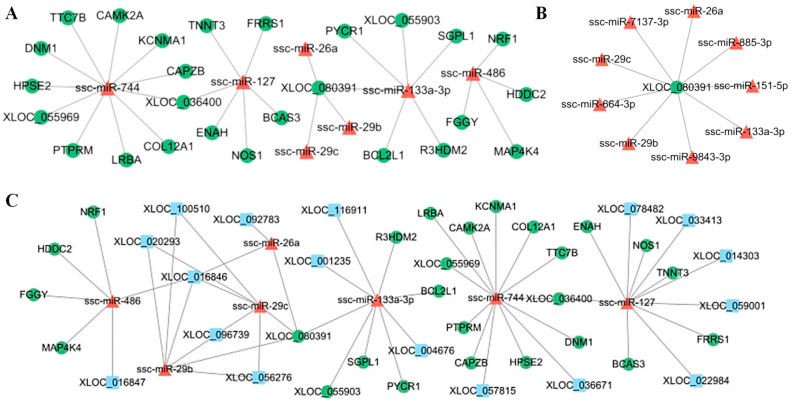
Visualization of the constructed interaction networks. (**A**) Visualization of the constructed miRNA–mRNA interaction network. (**B**) XLOC_080391-centered regulatory network. (**C**) Visualization of the constructed lncRNA–miRNA–mRNA interaction network. Red triangular nodes represent miRNAs, green circular nodes represent mRNAs, and blue square nodes represent lncRNAs.

In order to find potential regulatory networks, based on the correlation analysis of sequencing data and website prediction, we identified negative regulatory interactions between miRNAs and mRNAs that might act on muscle development (Table S4). Part of the miRNA-mRNA regulatory network was revealed (Figure 5A). Among the identified negative regulatory interactions, we found that seven miRNAs (ssc-miR-744, ssc-miR-127, ssc-miR-133a-3p, ssc-miR-26a, ssc-miR-29b, ssc-miR-29c, and ssc-miR-486) targeted multiple mRNAs. For example, ssc-miR-486 targeted *NRF1*, *HDDC2*, *FGGY*, and *MAP4K4*. Moreover, the down-regulated mRNA *XLOC_080391* was repressed by multiple DE miRNAs, including ssc-miR-133a-3p, miR-885-3p, ssc-miR-29b, ssc-miR-29c, and ssc-miR-7137-3p (Figure 5B). A negative regulatory network of lncRNA–miRNA was identified (Figure S2). Within this, 17 DE lncRNAs were considered to play a key role in regulatory networks.

The lncRNA–miRNA and miRNA–mRNA interactions identified in the above analyses have been combined to construct a composite lncRNA–miRNA–mRNA network (Figure 5C). This network consists of 50 nodes, including 7 miRNAs, 17 lncRNAs, and 26 mRNAs, which could be involved in ceRNA regulatory processes during muscle growth and fusion. Some of the mRNAs in this network—such as *TNNT3*, *NOS1*, and *ENAH*—have been known to be associated with muscle biology. We also observed that five lncRNAs were interrelated with ssc-miR-127, and may therefore act as ceRNAs to inhibit the translation of target genes. In addition, we found that XLOC_016847 may serve as a ceRNA to mediate *FGGY* and *NRF1* expression by sponging ssc-miR-486.

### 3.7. Validation of Sequencing Results via Quantitative Real-Time PCR (qRT-PCR)

**Figure 6:**
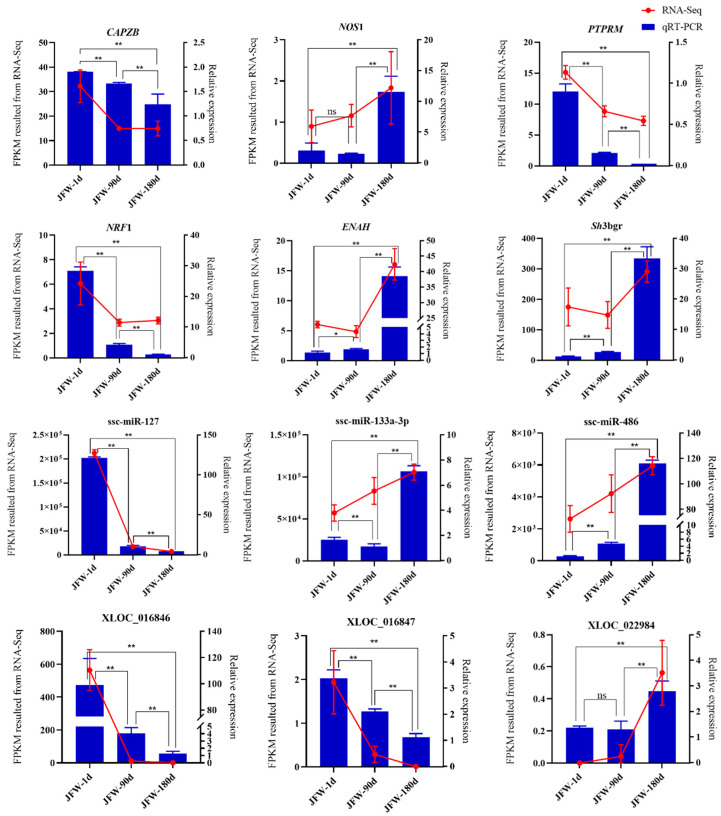
Expression levels of selected differentially expressed genes via qRT-PCR (blue), showing comparisons with the RNA-Seq data (red). *: significant difference (*p* < 0.05), **: extremely significant difference (*p* < 0.01), ns: the difference was not significant.

From the lncRNA–miRNA–mRNA interaction network, 12 key nodes were selected for validation via qRT-PCR, including six DE mRNAs, three DE miRNAs, and three DE lncRNAs. It was suggested that the results of qRT-PCR and RNA-seq showed a similar trend with an increase in age, and the expression trend of some miRNA-mRNA-lncRNA was in line with the ceRNA hypothesis (Figure 6). Thus, these differentially expressed mRNAs, miRNAs, and lncRNAs may contribute to the development of skeletal muscle. Together, these results demonstrate consistent expression patterns for the 12 differentially expressed genes across qRT-PCR and RNA-Seq, confirming RNA-Seq’s accuracy and reliability.

### 3.8. Bioinformatics Predicts Key Nodes of the Network

**Figure 7:**
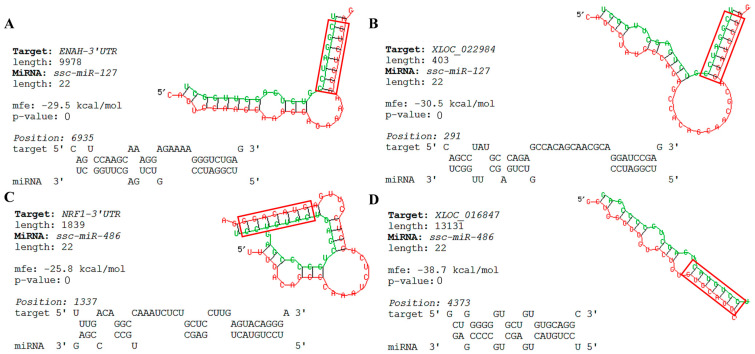
The secondary structure map of the binding site of the key nodes of the network. (**A**) The secondary structure map of the binding site between miR-127 and ENAH-3′UTR. (**B**) The secondary structure map of the binding site between miR-127 andXLOC_022984. (**C**) The secondary structure map of the binding site between miR-486 and NRF1-3′UTR. (**D**) The secondary structure map of the binding site between miR-486 and XLOC_016847. Green represents miRNA sequences, and red represents mRNA sequences.

The key nodes showing negative regulation in the network were further verified using RNAhybrid. The results showed that ENAH-3′UTR could bind to the “seed sequence” of miR-127 (Figure 7A). XLOC_022984 was also found at binding sites with the “seed sequence” of miR–127 (Figure 7B). The above results show that the expression of *ENAH*, XLOC_022984 was opposite to that of miR-127; *ENAH*/miR-127/XLOC_022984 may act as a ceRNA network involved in skeletal muscle development. It was also found that the NRF1-3′UTR and XLOC_016847 could bind to the “seed sequence” of miR-486 (Figure 7C,D). Their expression trends were consistent with the ceRNA hypothesis and may also act as a ceRNA network to regulate skeletal muscle growth and development.

## 4. Discussion

Since the introduction of sequencing technology, there has been a significant emphasis on evaluating the accuracy of various sequencing platforms. Each platform offers its own unique advantages and limitations. A study conducted on Illumina genome analyzers revealed a correlation between errors and specific sequence motifs that appeared prior to the error site [35,36]. Additionally, substitution bias was observed, with a clear preference for T substitution. Standardized experimental procedures, optimized sample preparation, and library construction can minimize errors during the preparation process. The implementation of strict quality-control measures and filtration steps before sequencing can eliminate low-quality readings and potential contamination, while repeated sequencing can help mitigate random errors.

In this study, we investigated nine cDNA libraries derived from LDM samples from Jinfen White pigs in the JFW_1d, JFW_90d, and JFW_180d. As a result, 2638 DE mRNAs and 982 DE lncRNAs were identified. These targets were enriched in a variety of pathways related to skeletal muscle growth and development. For example, the hippo signaling pathway has received much attention in recent years; it can regulate the activity of the proteins yes-associated protein and transcriptional co-activator with PDZ-binding motif to control tissue growth [37]. The PI3K-Akt and hedgehog signaling pathways are also well known for their roles in skeletal muscle development [38,39]. In another study, AMPK mediates skeletal muscle transcriptional regulation by altering gene expression [40]. The latest study found that the changes in muscle fiber types in the longissimus dorsi muscle of Ningxiang pigs at 1 month of age, 3 months of age, 5 months of age, and 7 months of age were analyzed via whole transcriptome sequencing. The ceRNA regulatory network was constructed, and KEGG and GO analysis results showed that 40 known genes and 6 new genes were related to skeletal muscle development and muscle fiber type changes through the PI3K-Akt pathway [41]. There were many other metabolic pathways that were also enriched and, taken together, the enrichment results suggest that the genes we screened out may be functionally relevant to skeletal muscle growth and development.

Among the 2638 DE mRNAs identified in this study, some have been shown to be essential to muscles. In response to mechanical and metabolic stimulus, NO signaling controls the maintenance of skeletal muscle integrity and appropriate signaling mechanisms. Differences in *NOS1* subcellular distribution and mechanical load change can induce changes in NO production [42], and *NOS1* was identified here as a target of miR-127. A proteomic analysis of the LDM, comparing Large White pigs and Meishan pigs, revealed that the F-actin-capping protein subunit beta (CAPZB) protein showed quite different expression profiles between breeds; it seems to be a candidate gene for meat production traits [43]. *PTPRM* was previously investigated in five different pig breeds and was found to regulate many cellular processes, including cell growth and differentiation, as a signaling molecule [44]. Importantly, we observed that *PTPRM* and *CAPZB* were highly expressed in the early stages of skeletal muscle development, providing additional evidence that these genes might play an important role in the early stages of muscle development.

Additionally, *NRF*1 has been shown to be involved in many disease processes [45,46], as well as being key to cholesterol homeostasis [47]. Additionally, Rovito characterized *NRF1* as a key player in muscle-specific enhancer–promoter communication that orchestrates myofiber size regulation [48]. EVH1-domain-containing proteins are essential in actin dynamics and muscle formation [49]. ENAH, a member of this class of proteins, is involved in somite development [50], and ENAH has been discovered to be a target of miR-127 in this study. A study showed that *Sh3bgr* regulates skeletal muscle development by cooperating with *ENAH* [51]. Here, the expression trends of *Sh3bgr* and *ENAH* were parallel and showed an increase with age, also indicating that these factors may interact to participate in skeletal muscle development. Taken together, the above considerations also suggest that the results are valuable in skeletal muscle composition and development.

The seven miRNAs screened in this study have already been implicated in muscle function. Many previous reports have established that miR-133a-3p is specifically expressed in porcine skeletal muscle [52,53]. The transfection of miR-133a-3p mimics into myotubes was found to induce muscle growth and also to regulate the Akt/mTOR/S6K signaling pathway by inhibiting the expression of target genes [54]. Other research showed that mice with miR-127 transgenes experienced significantly faster muscle regeneration than mice of the wild type [55]. Meanwhile, an investigation into the miRNA transcriptome in the LDM of Rongchang pigs during weaning and slaughter found that the overexpression of miR-127 inhibited the proliferation of porcine skeletal muscle satellite cells and myotube fusion [56]. This result was in contrast with the observed effects of miR-127 in C2C12 mouse myoblasts, indicating that the functions of miR-127 are species-dependent, and the mechanisms involved may also be different. In this study, the expression of miR-127 decreased with age, suggesting that it may be involved in the regulation of skeletal muscle development.

Previous works have also found a dramatically altered expression of miR-744 in Qinchuan bovine muscle tissue with age. There was a significant increase in miR-744 expression in the skeletal muscle of Qinchuan cattle during embryonic development compared with age-old animals [57]. Furthermore, miR-486 is one of the intermediate molecules in the myostatin and IGF-1/Akt/mTOR pathways, which regulate skeletal muscle size [58]. The transcription of miR-486 is, in turn, directly controlled by *SRF*, *MRTF-A*, and *MyoD*, of which it can be regarded as a downstream mediator [59]. Thus, both of these miRNAs (miR-744 and miR-486), which were identified as part of the interaction network in this study, may be potential molecular markers with important roles in muscle development.

By constructing lncRNA–miRNA–mRNA regulatory networks using bioinformatics, we also identified a number of lncRNAs of interest. As an important class of non-coding RNA, lncRNAs exist widely in various organisms, with functions involved in various aspects, such as the cell cycle and individual development [60,61]. In recent studies, it has been shown that lncRNAs are capable of regulating gene expression in three different ways: epigenetic, transcriptional, and post transcriptional. Previously, porcine LDM expression profiles have been constructed using RNA-Seq analysis at different developmental stages [62]. Additionally, we examined the number of exons and the chromosomal distribution of the identified lncRNAs, as well as their expression levels. About 35% of them were derived from chromosome 1, containing only one or two exons [63]. These findings are consistent with previous research.

The study of lncRNAs has advanced significantly over the last decade, but only a small number of annotated lncRNAs have been extensively studied. But hypotheses about the general mechanism of lncRNA function have also been initially explored. In particular, the mechanism of lncRNAs acting as ceRNAs has attracted much attention [64]. For example, recent studies have found that lncMUMA regulates *MyoD* expression by binding to miR-762 to promote myoblast differentiation. Enhanced expression of lncMUMA can rescue the decrease in *MyoD* and muscle mass caused by miR-762 knockout [65]. Further, H19 is one of the best-known long noncoding RNAs, and it plays a key role in the differentiation of skeletal muscle [66,67]. By competitively binding to miR-140-5p, H19 inhibits the differentiation of porcine skeletal muscle satellite cells [68]. In our analysis, we screened several ceRNA regulatory networks and identified multiple target genes and novel lncRNAs centered on seven miRNAs. These lncRNAs may, therefore, act as ceRNAs to competitively bind miRNAs and regulate the expression of downstream target genes. Further, qRT-PCR verified that the expression trend of miR-127 was opposite to that of *NOS1*, *ENAH*, and XLOC_022984. The expression trend of miR-486 was inversely correlated with those of *NRF1*, XLOC_016846, and XLOC_016847. Thus, these lncRNAs (XLOC_016846, XLOC_016847, and XLOC_022984) may be involved in the regulation of skeletal muscle development through the ceRNA mechanism.

## 5. Conclusions

In conclusion, we constructed the transcriptome profile of the longissimus dorsi muscle of Jinfen White pigs at 1d, 90d, and 180d. A total of 2638 differentially expressed DE mRNAs and 982 differentially expressed DE lncRNAs were identified. The DE mRNAs were target genes of DE lncRNAs enriched in multiple pathways related to skeletal muscle growth and development, such as the PI3K-Akt, hedgehog, and hippo signaling pathways. We also built a critical ceRNA regulatory network that included 7 miRNAs, 17 lncRNAs, and 26 mRNAs. Among them, XLOC_022984/miR-127/*ENAH* and XLOC_016847/miR-486/*NRF*1 may act as key ceRNA networks to regulate skeletal muscle function. Thus, the present findings will provide a useful and convenient resource for further studies on muscle development and molecular breeding in pigs.

## Figures and Tables

**Table 1:** Summary of data from RNA-Seq.

Samples	Raw Reads	Clean Reads	Q20 (%)	Q30 (%)	GC Content (%)
JFW_1d_1	92,655,706	91,942,766	98.62	95.72	55.62
JFW_1d_2	107,050,232	106,444,968	98.64	95.78	57.33
JFW_1d_3	104,392,040	103,814,406	98.58	95.45	47.55
JFW_90d_1	96,202,328	95,550,798	98.08	94.20	55.77
JFW_90d_2	106,311,518	105,563,842	98.56	95.75	60.57
JFW_90d_3	95,622,436	94,984,938	98.67	95.89	56.13
JFW_180 d_1	94,853,354	94,375,978	97.81	93.78	57.86
JFW_180 d_2	104,189,042	103,407,232	98.59	95.54	54.04
JFW_180 d_3	107,380,946	106,567,824	98.40	95.04	55.09

## Data Availability

The original data for the RNA-seq data were submitted to the SRA Database (BioProject ID: PRJNA867525).

## Supplementary Materials

The following supporting information can be downloaded at: https://www.mdpi.com/article/10.3390/cimb46010022/s1, Figure S1: miRNA-lncRNA regulatory networks; Figure S2: Prediction of lncRNA coding ability; Table S1: Primers of RT-qPCR; Table S2: The functional enrichment results of DE mRNAs. Table S3: The target genes and functional enrichment results for DE lncRNAs. Table S4: miRNA-mRNA regulatory networks.
