# Supplementary material for: Construction of LncRNA-Related ceRNA Networks in Longissimus Dorsi Muscle of Jinfen White Pigs at Different Developmental Stages
Source: Curr Issues Mol Biol. 2024 Jan 2;46(1):340–54. doi: 10.3390/cimb46010022 (PMC10814722; doi:10.3390/cimb46010022)
Supplement: Supplementary file 1 [file cimb-46-00022-s001.zip › Supplementary file Table S1.pdf]

Table S1 Primers of RT-qPCR.

mRNAs, miRNAs, lncRNAs	Primer sequence (5′ -3′)	Prouduct Length
CAPZB	F: TGACCCGGATGTTCACTCCT<br>R: TCAAGTCCAAGGCACAGTCC	145 bp
*NOS*1	F: GGGGATAGTCCCCACTAGCA<br>R: TGGCATGATTTCCTGCGACT	187 bp
PTPRM	F: TGCTGTTGAAAAGGGAGGGG<br>R: CACCTCGCTGTGACACTTCT	71 bp
*NRF*1	F: GAAGCTGTCCAGGGGCTTTA<br>R: ATCCATGCTCTGCTACTGGG	116 bp
ENAH	F: CCCCTCCCCAGCTTTTCTTT<br>R: ACAAGTGTCCACCTCCTCCT	118 bp
*Sh*3bgr	F: ATTCCTCTGCCTCCCCAGAT<br>R: TTCCTCGGGCGATTTTGTCA	147 bp
ssc-miR-127	F: GGGTCGGATCCGTCTGAGC<br>R: CAGTGCGTGTCGTGGAGT	67 bp
ssc-miR-133a-3p	F: GGGTTGGTCCCCTTCAAC<br>R: CAGTGCGTGTCGTGGAGT	62 bp
ssc-miR-486	F: GGGTCCTGTACTGAGCTGC<br>R: CAGTGCGTGTCGTGGAGT	61 bp
XLOC_016846	F: GCTTGGAGGGACATGACACA<br>R: TGTCGTTTGAGGGTTCTGGG	184 bp
XLOC_016847	F: CATGTTTGCAGGCGTGTTCA<br>R: GGCAAGCAAAGTGGACAGTG	135 bp
XLOC_022984	F: CATTTAGTGAGGACTTGGGGCT<br>R: GGTTACTGTTCTTGTTTAACACTCC	70 bp
18s RNA	F: ATGCCAGAGTCTCGTTCGTTAT<br>R: CGGACAGGATTGACAGATTGAT	119 bp
U6	F: CTCGCTTCGGCAGCACA<br>R: AACGCTTCACGAATTTGCGT	60 bp
