# Supplementary figures and images for: Construction of LncRNA-Related ceRNA Networks in Longissimus Dorsi Muscle of Jinfen White Pigs at Different Developmental Stages
Source: Curr Issues Mol Biol. 2024 Jan 2;46(1):340–54. doi: 10.3390/cimb46010022 (PMC10814722; doi:10.3390/cimb46010022)

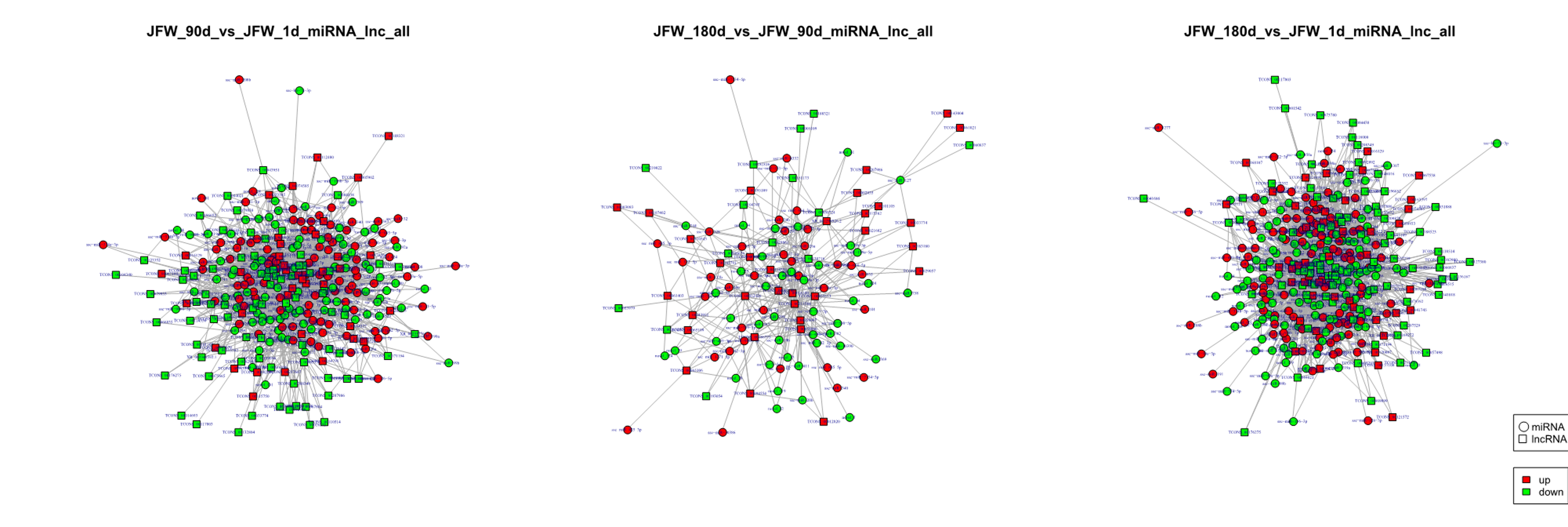

Supplement: Supplementary file 1 [file cimb-46-00022-s001.zip › Supplementary file Figure S1.png]

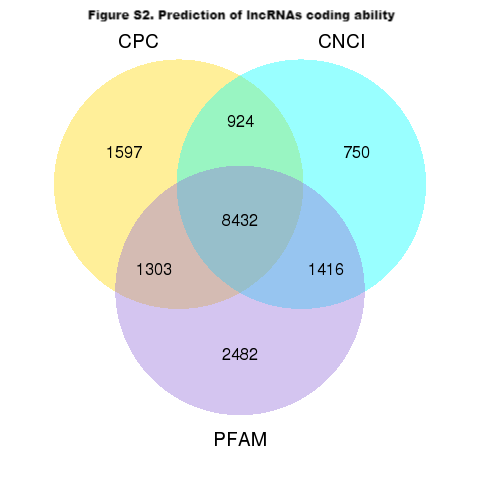

Supplement: Supplementary file 1 [file cimb-46-00022-s001.zip › Supplementary file Figure S2.png]
